# The Pga59 cell wall protein is an amyloid forming protein involved in adhesion and biofilm establishment in the pathogenic yeast *Candida albicans*

**DOI:** 10.1038/s41522-023-00371-x

**Published:** 2023-01-25

**Authors:** Thierry Mourer, Mennat El Ghalid, Gérard Pehau-Arnaudet, Brice Kauffmann, Antoine Loquet, Sébastien Brûlé, Vitor Cabral, Christophe d’Enfert, Sophie Bachellier-Bassi

**Affiliations:** 1 Institut Pasteur, Université Paris Cité, INRAE USC2019, Fungal Biology and Pathogenicity, F-75015 Paris, France; 2 Institut Pasteur, Université Paris Cité, CNRS UMR 3528, Plateforme de Bio-imagerie Ultrastructurale, F-75015 Paris, France; 3 Univ. Bordeaux, CNRS, INSERM, IECB, UAR 3033, Pessac, France; 4 Univ. Bordeaux, CNRS, Bordeaux INP, CBMN, UMR 5248, IECB, Pessac, France; 5 Institut Pasteur, Université Paris Cité, CNRS UMR 3528, Plateforme de Biophysique Moléculaire, F-75015 Paris, France; 6 Present Address: Instituto Gulbenkian de Ciência, Oeiras, Portugal

**Keywords:** Biofilms, Pathogens

## Abstract

The human commensal fungus *Candida albicans* can attach to epithelia or indwelling medical devices and form biofilms, that are highly tolerant to antifungal drugs and can evade the immune response. The cell surface protein Pga59 has been shown to influence adhesion and biofilm formation. Here, we present evidence that Pga59 displays amyloid properties. Using electron microscopy, staining with an amyloid fibre-specific dye and X-ray diffraction experiments, we showed that the predicted amyloid-forming region of Pga59 is sufficient to build up an amyloid fibre in vitro and that recombinant Pga59 can also adopt a cross-β amyloid fibre architecture. Further, mutations impairing Pga59 amyloid assembly led to diminished adhesion to substrates and reduced biofilm production. Immunogold labelling on amyloid structures extracted from *C. albicans* revealed that Pga59 is used by the fungal cell to assemble amyloids within the cell wall in response to adhesion. Altogether, our results suggest that Pga59 amyloid properties are used by the fungal cell to mediate cell-substrate interactions and biofilm formation.

## Introduction

Fungal infections have emerged over the past decades as a serious threat for human health^1–3^. Among all human fungal pathogens, *Candida albicans* is the most frequently encountered species in hospitals. Though a normal resident of the human gastrointestinal or genital tracts, *C. albicans* can under certain circumstances proliferate and cause superficial benign infections such as oral or vaginal thrush. However, the combination of local factors (broad-spectrum antibiotics), underlying diseases (chemotherapy-induced mucosal erosion, neutropenia) and invasive procedures (abdominal surgery, central venous catheter) favours translocation of *C. albicans* across the mucosa and dissemination into the bloodstream^4^. Recent epidemiological surveys have reported that invasive candidiasis accounts for 75% of all invasive fungal infections^5–7^ and *Candida* species are now recurrently found among the five leading causes of healthcare-associated bloodstream infections^8–10^. The invasive candidiasis mortality rate (>40%) in immunocompromised patients is higher than that of any bacterial sepsis^11–13^. Because of the increase of hospitalised patients with serious underlying diseases, especially those undergoing treatment for haematological malignancies or hospitalised in ICUs, systemic candidiasis has become a life-threatening challenge for human health.

*C. albicans* is well known for its ability to form biofilms on medical devices, a process that contributes to relapse or persistence of candidiasis despite antifungal treatments^14^. Indeed, *C. albicans* biofilms are crucial for pathogenesis by reducing the sensitivity of the fungus to both antifungal agents and the host immune system^11^. The molecular processes allowing *C. albicans* biofilms to avoid antifungal toxicity are mechanistically complex. The resistance towards antifungal drugs is largely due to their trapping by the biofilm extracellular matrix (ECM) and, to some extent, the upregulation of efflux pumps^15^. Upregulation of both ABC and MFS transporters contributes to the cellular export of antifungal components and thus avoids their toxicity. Biofilms are embedded in the ECM, which protects them from environmental insults, creating a steric hindrance that prevents drug penetration inside the biofilm^16^. In addition, ergosterol production is decreased in mature biofilm as compared to planktonic cells rendering the microbial community less susceptible to azoles and polyenes than planktonic cells^17^. The acquisition of new knowledge on the establishment of fungal biofilm will allow the development of innovative strategies to counter the threat posed by these microbial communities.

Biofilm formation is a process tightly regulated in *C. albicans* that occurs in response to several environmental clues such as surface contact, CO_2_ levels or nutrient starvation^18–20^. This biological process is orchestrated by a « core » of nine transcription factors that finely regulate lipid metabolism, filamentation and above all adhesion^21,22^. Adhesion of *C. albicans* to a substrate is mediated by several polypeptides that belong to three families of adhesins: Als, Hwp1 and Iff/Hyr^23^. Some proteins among these families, namely Als1, Als2, Als3, Als5, Hwp1, Hwp2 and Eap1, were described to impact biofilm formation^23^. Except for Als2, all adhesins of *C. albicans* involved in biofilm establishment contain β-aggregation-prone sequences suggesting that they may be assembled as amyloid structures^24–26^. Amyloid fibres correspond to filamentous protein aggregates with an organised structure. The assembly of an amyloid fibre usually consists of two steps: first, amyloids are nucleated by the homomultimerisation of a protein through the amyloid-forming region. Subsequently, the amyloid fibre is elongated by the stacking of hundreds of monomers on top of each other. Structurally, amyloid fibres are characterised by a high β-sheet content as well as by the presence of the typical cross-β arrangement, which is a hallmark of such quaternary structures^27^. The cross-β structure is characterised by two reflections detected at 4.7 Å and ~10 Å by X-ray diffraction, which respectively corresponds to the inter-strand and inter-sheet spacing^27^.

Amyloid structures have long been thought to be harmful to living organisms. However, recent reports have demonstrated that some proteins in the amyloid form can acquire new properties that could be useful and beneficial in the cellular context, a phenomenon called functional amyloids^28^. For instance, the curli system in *E. coli* consists of amyloid fibres using the proteins CsgA and CsgB as substrates. These fibres extended in the extracellular matrix of the bacteria are involved in biofilm formation and surface colonisation^29^. Another example is the *S. cerevisiae* pyruvate kinase, Cdc19, which forms amyloid fibres aggregating into stress granules under different types of stress. This phenomenon protects the protein from degradation and facilitates the resumption of the cell cycle once the stress is over^30^.

Results based on electron microscopy, as well as heterologous expression of *C. albicans* proteins in *S. cerevisiae*, have revealed that Als1, Als3, Als5 and Eap1 genuinely adopt an amyloid-like structure^22,23^. Over the last decade, knowledge gathered mainly on Als1 and Als5 from *C. albicans* has tended towards a model in which these adhesins are unfolded by shear forces generated by liquid flow^25^, unmasking the amyloid-forming regions and allowing lateral and homotypic amyloid interactions to occur in the *C. albicans* cell wall, thus creating a very dense area of adhesins, called amyloid nanodomain^31,32^. Finally, these nanometre-sized nanodomains create adhesive patches on the cell surface, enabling cell–cell interactions through adhesins Ig-like domain^32^. Pga59 is another adhesin found in *C. albicans* that belongs to the Hwp1 family. Originally, Pga59 was identified by a computational screen that aimed to find all GPI-anchored proteins in several fungi species, including *C. albicans*^33^. The proteins found in the in silico analysis, with no functional name, were given the acronym Pga for predicted GPI-Anchored protein associated with a number i.e. Pga59^33^. Pga59 is a cell wall-associated protein harbouring both N- and O-glycosylation that was originally described to maintain cell wall integrity in *C. albicans*^34^. Previous results from our laboratory have shown that *PGA59* is highly expressed in *C. albicans* biofilms^34^, and that overexpression of *PGA59* has a positive impact on biofilm formation by increasing the dry weight of the microbial community^35^. Furthermore, atomic force microscopy experiments have revealed that *PGA59* overexpression is also associated with higher adhesion forces on the cell surface, resulting in increased adhesion to a plastic substrate upon *PGA59* upregulation^35^. However, the molecular mechanisms underlying Pga59 function in biofilm formation remain to be understood.

In this study, we have shown that Pga59 is assembled into amyloid structures upon adhesion, and we have demonstrated that Pga59 amyloid formation has a positive impact on cell adhesiveness as well as on biofilm establishment in *C. albicans*. In addition to the work on Als proteins, our study provides further strong evidence that amyloid structures play a role in fungal cell adhesion to a substrate. Furthermore, we describe for the first time that Pga59-based amyloid structures have a direct impact on biofilm formation in *C. albicans*, and thus constitute a promising avenue for the development of new antifungal strategies.

## Results

### The D1 domain of Pga59 triggers β-amyloid assembly in vitro

**Fig. 1:**
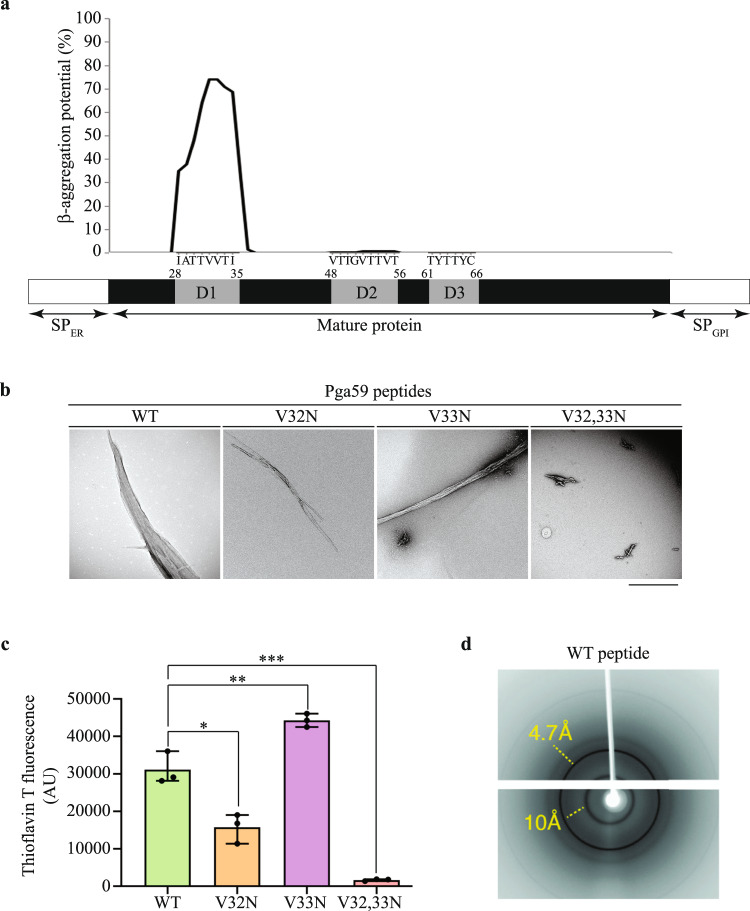
The D1 domain of Pga59 is sufficient to trigger amyloid fibre assembly in vitro. **a** The graph shows the TANGO β-aggregation potential of each amino acid in the Pga59 mature region. The amino acid composition of the amyloid-forming regions D1, D2 and D3 is indicated below the graph. Pga59 is sketched under the graph. The black rectangle represents Pga59 full-length protein, and the numbers above indicate the amino acid position encompassing D1, D2 and D3 domains. The SP_ER_ and SP_GPI_ regions correspond to the signal peptides for translocation into the endoplasmic reticulum (ER) and anchorage to a preformed GPI lipid attached to the ER membrane, respectively. The grey boxes named D1, D2, and D3 represent the amyloid-forming domains in Pga59 mature region predicted by TANGO. **b**, **c** Wild type and mutant peptides of Pg59 were resuspended in DMSO prior to their dilution in Tris buffer. After 16 h at 37 °C, peptides were analysed with transmission electron microscopy and ThT staining. **b** D1 peptides were dropped on electron microscopy grids and negatively stained with uranyl acetate. Peptides’ characteristics are indicated above each micrograph. Scale bar: 500 nm. **c** ThT intensity of each peptide was recorded at 496 nm. The histogram shows the quantification of data from three independent experiments. Values are displayed as the averages ± *SD*. (*) *p* < 0.05, (**) *p* < 0.01, (***) *p* < 0.001. The significance of the ThT fluorescence differences were tested using Student’s *t*-test. **d** X-ray diffraction pat*t*ern of the quaternary structures formed by the WT D1 peptide. Source data are provided as a Source Data file.

Using the in silico AMYLPRED2 tool^36,37^, we identified three potential amyloid-forming domains in the Pga59 mature sequence, D1 from position 28 to 35 (^28^IATTVVTI^35^), D2 from 48 to 56 (^48^VTTGVTTVT^56^) and D3 from 61 to 66 (^61^TYTTYC^66^) (Fig. 1a). Further analysis with TANGO, one of the programmes run by AMYLPRED2, showed that D1 was the only domain presenting a high β-aggregation potential (Fig. 1a). Within D1, the highest β-aggregation potential (~74%) was predicted for two valines at positions 32 and 33 (Fig. 1a). A synthetic eight amino acid-long peptide overlapping this sequence (residues 28 to 35) was obtained, as well as peptides incorporating mutations of either V32 or V33 or both into asparagine residues (V32N, V33N and V32,33N, respectively). To confirm the amyloidogenic properties of the D1 sequence, transmission electron microscopy (TEM) observation of the negatively stained synthetic peptides was performed after incubation at 37 °C for 16 h. The WT peptide of the D1 domain formed a large micrometre-sized aggregate with organised regions exhibiting a fibrillar morphology (Fig. 1b). The single mutant peptides V32N and V33N were still able to adopt a fibrillar morphology reminiscent of an amyloid fibre. However, the peptide in which both valines had been substituted for asparagines was unable to adopt an elongated fibrillar morphology (Fig. 1b). To determine if the fibrillar aggregates observed in TEM corresponded to amyloid structures, we performed in vitro Thioflavin T (ThT) assays on the same set of synthetic peptides. The WT peptide displayed a very high fluorescence intensity (~30k arbitrary units (AU)) generated by amyloid structures stained with ThT (Fig. 1c). The V32N mutation had a limited impact, reducing the fluorescence intensity by twofold to ~15k AU while the V33N mutation enhanced it by 1.4-fold to ~44k AU (Fig. 1c). However, the fluorescence intensity of the V32,33N peptide upon ThT staining was significantly reduced to a value of 987 AU, i.e. more than 30 times less than to the WT (Fig. 1c). As the WT D1 peptide of Pga59 self-assembles in fibrillar quaternary structures that are positive to ThT, we sought to determine if these aggregates are genuine amyloids exhibiting a typical cross-β signature. Hence, we used X-ray fibre diffraction to investigate the amyloid nature of the D1 WT peptide. The X-ray diffraction pattern showed two main reflections at 4.7 and 10 Å (Fig. 1d). Such a pattern is characteristic of the so-called cross-β arrangement, one of the structural hallmarks of amyloid fibrils^27^. This clearly indicates that the Pga59 D1 domain is assembled into amyloid fibres made of β-strands in its aggregated state. The cross-β pattern reveals a repetition of β-strands that are parallel to the main fibril axis and separated by 4.7 Å. The β-sheets are perpendicular to the fibril axis with a spacing of 10 Å. Taken together, these results revealed that the D1 domain of Pga59 is sufficient to craft a cross-β amyloid fibre and that the presence of valine at positions 32 and 33 is critical for this process.

### Recombinant full-length Pga59 is assembled as an amyloid structure

**Fig. 2:**
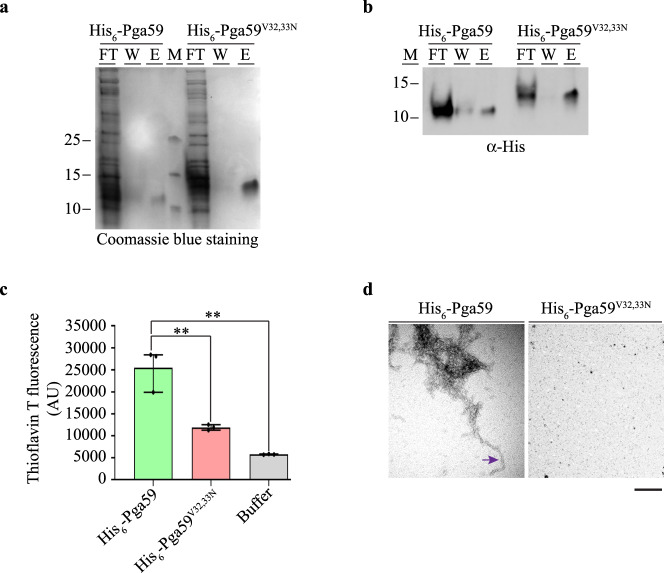
rPga59 aggregates display amyloid structures properties. HIS_6_-Pga59 and HIS_6_-Pga59^V32,33N^ proteins were expressed in *Escherichia coli* and purified by affinity chromatography. rPga59 proteins were analysed with Coomassie blue staining (**a**) and western blot using an α-His antibody (**b**). Fractions loaded on the gel correspond to the flow through (FT), the wash (W) and the elution (E). (M): Molecular weight markers. Sizes are indicated on the left. **c** ThT fluorescence intensity of each recombinant proteins analysed at 496 nm. The histogram shows the quantification of data from three independent experiments. Values are displayed as the averages ± SD. (**) *p* < 0.01. The significance of the ThT fluorescence differences was tested using Student’s *t*-test. **d** Wild type (left) and mutated (right) rPga59 were negatively stained with uranyl acetate and analysed with transmission electron microscopy. An isolated amyloid fibre formed by the WT protein is pinpointed on the micrograph with an arrow. Scale bar: 100 nm. Source data are provided as a Source Data file.

We next examined if the ability of the D1 domain to form an amyloid structure is conserved with the full-length Pga59 protein. To do so, we produced Pga59 and Pga59 ^V32,33N^ (in which both valine residues were replaced by asparagines) fused to an N-terminal His-tag (His_6_-Pga59 and His_6_-Pga59^V32,33N^, respectively) in *Escherichia coli* SHuffle T7 and purified the recombinant Pga59 proteins (rPga59) by affinity chromatography. We chose to work with the double mutant since the amyloid assembly was blocked only in the D1^V32,33N^ peptide (Fig. 1b, c). Both versions of the rPga59 were purified from bacterial inclusion bodies (Fig. 2a) and their purity was assessed by MALDI-TOF mass spectrometry. For His_6_-Pga59, the mass analysis revealed the presence of a single intense peak at 9, 839 Da, which corresponds to the expected mass of the WT recombinant protein. Regarding His_6_-Pga59^V32,33N^, a single intense peak was observed at the expected mass of 9, 869 Da (Supplementary Fig. 1). The mass analysis demonstrates that both WT and mutant rPga59 have been purified to a high degree. This result allows us to propose that the gel migration of His_6_-Pga59^V32,33N^, noticeably slower than that of His_6_-Pga59, is most likely due to charge variations affecting the migration of the mutant protein compared to the WT. Subsequently, a single signal was detected in the elution fraction by an α-His antibody, confirming that polypeptides eluted from the affinity chromatography matrix were genuinely either His_6_-Pga59 or His_6_-Pga59^V32,33N^ (Fig. 2b). To evaluate the ability of rPga59 to adopt an amyloid shape, we conducted ThT assays and TEM. Following refolding, both the WT and the mutant version of rPga59 were incubated at 37 °C and subsequently either treated with ThT or negatively stained with uranyl acetate and observed by electron microscopy. His_6_-Pga59 displayed a strong ThT signal (~25k AU, Fig. 2c) and adopted elongated fibrillary structures (purple arrow, Fig. 2d). On the other hand, His_6_-Pga59^V32,33N^ produced a 2.5-fold weaker ThT signal (~10k AU) and no fibrillary aggregates were observed (Fig. 2c, d). These results suggest that the amyloid assembly capabilities of His_6_-Pga59^V32,33N^ are greatly reduced compared to His_6_-Pga59. X-ray diffraction was also used to confirm the amyloid nature of His_6_-Pga59, and typical signatures of β-amyloid fibre were observed for His_6_-Pga59, with an X-ray diffraction pattern showing reflections at 4.7 and 10 Å (Supplementary Fig. 2). In conclusion, ThT staining, TEM and X-ray diffraction measurements demonstrate that *E. coli-*produced Pga59 is assembled as a typical cross-β amyloid structure in vitro.

### Heterologous expression of Pga59 triggers amyloid structure assembly in the budding yeast cell wall

**Fig. 3:**
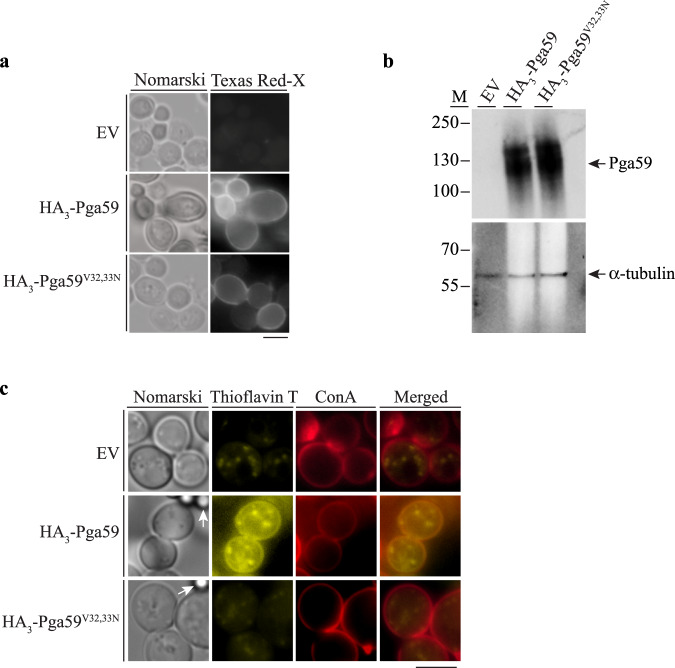
Heterologous expression of Pga59 in the budding yeast leads to amyloid structures assembly in the cell wall. **a** *S. cerevisiae* strains expressing HA_3_-Pga59, HA_3_-Pga59^V32,33N^ or the empty vector as a negative control (EV), were cultivated in a YPD medium. The cellular location of HA_3_-Pga59 and HA_3_-Pga59^V32,33N^ upon cell adhesion was assessed by immunofluorescence microscopy using an α-HA antibody. Right panels: Fluorescent signals observed with an α-mouse Texas Red-X conjugate as a secondary antibody. Left panels: Nomarski. **b** Pga59 levels were assessed in each genetic background by western blot (α-HA antibody, top) using α-tubulin (bottom) as a loading control. The chemiluminescent signals were obtained using a monoclonal α-mouse HRP secondary antibody. Left: Molecular weight standards (M). **c** amyloid assembly was assessed in the indicated strains by ThT staining (yellow channel) upon adhesion triggered by BSA-coated magnetic beads (arrows in the left panels). The cell wall of *S. cerevisiae* was stained with the Concanavalin A, Alexa Fluor 594 conjugate (red channel). Yellow and red channels were merged on the right part of the panel. Scale bar: 5 μm. The data presented here are representative of three independent experiments. Source data are provided as a Source Data file.

We next sought to determine whether amyloid fibre assembly of Pga59 is a biological process relevant in yeast. We first expressed HA_3_-Pga59 and HA_3_-Pga59^V32,33N^ in *S. cerevisiae* to avoid any background due to amyloid production by other proteins in *C. albicans* such as Als1 or Als5, among others^23^. Using immunofluorescence staining, we showed that both the WT HA_3_-Pga59 and the HA_3_-Pga59^V32,33N^ mutant proteins were properly localised at the cell surface of *S. cerevisiae*, while no signal was observed in yeast cells expressing the empty vector (Fig. 3a). We also confirmed that both proteins were produced at similar levels (Fig. 3b). The molecular weight of Pga59 is higher when expressed in *S. cerevisiae* as protein glycosylation occurs in yeast and not in *E. coli*. We triggered cell adhesion by incubating *S. cerevisiae* cells expressing either HA_3_-Pga59 or HA_3_-Pga59^V32,33N^ with BSA-coated magnetic beads, and assessed the formation of amyloid structures using ThT staining in vivo. *S. cerevisiae* cells expressing HA_3_-Pga59 displayed a ThT-fluorescent signal at the cell surface, as indicated by colocalization with a cell wall marker, namely Concanavalin A, Alexa Fluor^TM^ 594 conjugate (Fig. 3c). In contrast, *S. cerevisiae* cells expressing either the empty vector or HA_3_-Pga59^V32,33N^ did not display a cell surface-localised ThT signal. Here, the ThT fluorescence was restricted to weak cytoplasmic foci that did not colocalize with the Concanavalin A signal (Fig. 3c). Altogether, these data suggest that Pga59 is involved in the assembly of amyloid structures in the fungal cell wall. Interestingly, the replacement of valines by asparagines at positions 32 and 33 in Pga59, shown above to impair the formation of amyloid structures in vitro, also prevents Pga59-induced assembly of parietal amyloid structures in *S. cerevisiae*.

### Amyloid structures formed upon adhesion in *C. albicans* involve Pga59

**Fig. 4:**
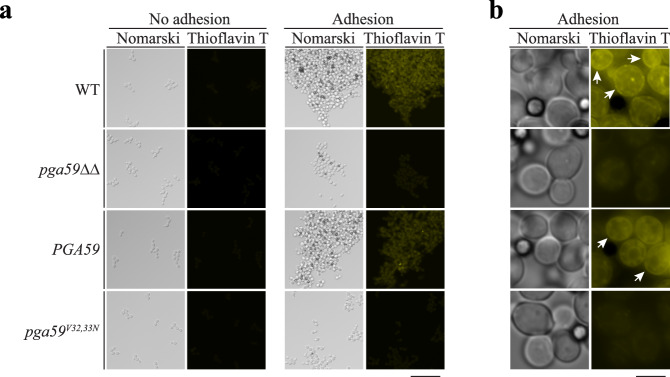
*C. albicans* assembly of amyloid structures upon adhesion requires Pga59. **a** Amyloid structures formation in *C. albicans* was monitored by using ThT staining. WT, *pga59Δ/pga59Δ*, *PGA59/pga59Δ* and *pga59*^*V32,33N*^*/pga59Δ* strains were cultured in a YPD medium. Adhesion was triggered by adding magnetic beads to the culture medium. Fungal cells without magnetic beads were used as a negative control (no adhesion, left panels). The ThT signal was recorded in the yellow channel while cells were observed by Nomarski (with a 20X objective). Scale bar: 25 μm. **b** Amyloid assembly was monitored with ThT staining (yellow channel) at higher magnification (with a 100X objective). Amyloid structures found in the cell wall are indicated with white arrows. Scale bar: 3 μm.

Based on the observations that Pga59 can self-assemble as amyloid fibres in vitro and trigger the formation of amyloid structures in the cell wall of *S. cerevisiae*, we aimed to assess the contribution of Pga59 in amyloidogenesis in *C. albicans*. To this aim, the WT strain, the *pga59ΔΔ* knockout and the two reintegrants *PGA59/pga59Δ* and *pga59*^*V32,33N*^*/pga59Δ* strains grown to mid-logarithmic phase were incubated with ThT and treated with BSA-coated magnetic beads to trigger adhesion^38^. Cells untreated with magnetic beads were used as a negative control for adhesion. Cells were then observed with an epifluorescence microscope to record the fluorescence generated by ThT bound on amyloid structures. Regardless of the genetic background, non-adherent cells (no magnetic beads) were poorly fluorescent, suggesting that amyloids are not formed in planktonic cells without adhesion. However, upon adhesion, Thioflavin T fluorescence dramatically increased in the WT, suggesting that amyloid structures are crafted in response to adhesion (Fig. 4a). In *pga59ΔΔ* mutant cells, there was a sharp reduction of the ThT fluorescence signal as compared to the WT (Fig. 4a), suggesting the involvement of Pga59 in the formation of adhesion-triggered amyloid structures. Accordingly, the reintegration of the *PGA59* WT allele in the knockout strain resulted in a strong ThT-fluorescent signal. In contrast, the reintegration of the mutant expressing the *pga59*^*V32,33N*^ allele did not restore a strong ThT-fluorescent signal, suggesting that this form of Pga59 is unable to trigger the production of cell surface amyloids in these conditions (Fig. 4a, right panels). We checked by RT-qPCR that *PGA59* and *pga59*^*V32,33N*^ are expressed at the same level, confirming that the phenotypes are not caused by differences in gene expression (Supplementary Fig. 3a). Further, we showed by western blot analysis and epifluorescence microscopy that the protein levels of GFP-Pga59 and GFP-Pga59^V32,33N^ were similar, and that both proteins were properly located at the cell surface, respectively (Supplementary Fig. 3b, c). Taken together, these results demonstrate that Pga59 is involved in amyloid assembly upon adhesion. Furthermore, the Pga59^V32,33N^ mutant protein that is unable to form amyloid fibres in vitro is also dysfunctional in vivo, possibly because of its inability to trigger amyloid structure formation in response to adhesion. To get a better insight into the cellular distribution of the amyloid structures, we observed the cells at a higher magnification (100X). The WT strain, as well as the reintegrant expressing the WT *PGA59* allele, displayed a diffuse ThT fluorescence signal in the cytoplasm as well as a strong signal at the cell surface (Fig. 4b). This ThT cell surface signal was dependent on the presence of a functional Pga59 as the knockout mutant, as well as the strain expressing the *pga59*^*V32,33N*^ allele, lost the ability to form cell surface amyloids (Fig. 4b). Results presented here clearly show that *C. albicans* produces cell surface amyloids upon adhesion in a Pga59-dependent manner.

### Pga59 is used by *C. albicans* to craft amyloid fibres

**Fig. 5:**
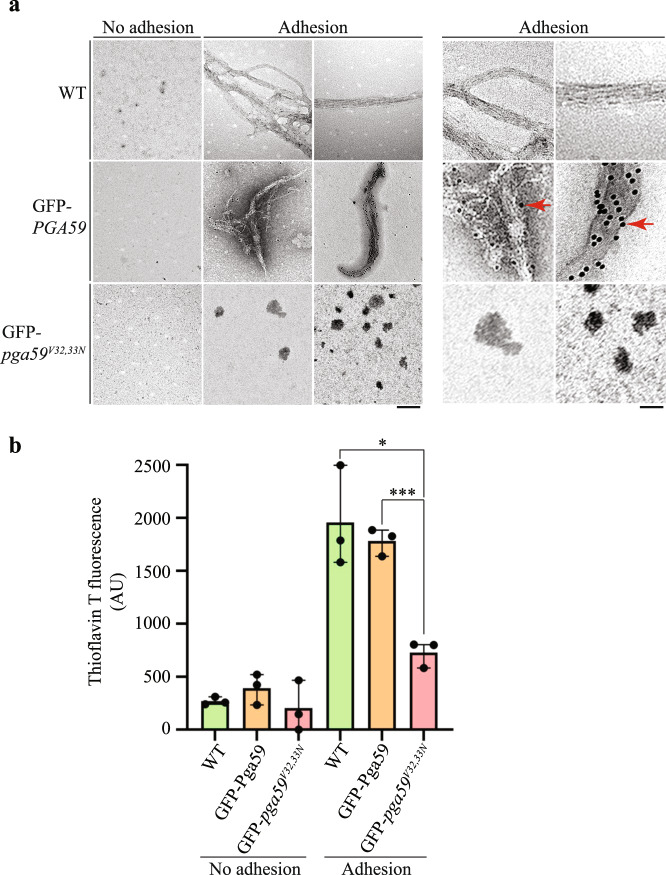
GFP-Pga59 is assembled within amyloid structures in adherent cells. **a** Adhesion of *C. albicans* strains expressing either GFP-Pga59, GFP-Pga59^V32,33N^ or the empty vector was triggered with magnetic beads and non-adherent cells were used as a negative control. Amyloid structures were enriched using a cell fractionation protocol developed to extract amyloids from yeast crude extracts. The resulting fibres were subsequently dropped on a copper grid, labelled with both the α-GFP antibody and gold particles prior to their negative staining with uranyl acetate. Micrographs are representative of the amyloid content in each condition. Scale bar: 200 nm. The right panels are a 2.5X enlargement of the adhesion columns from the left part (scale bar: 80 nm). Pga59 positive fibres are indicated with red arrows (right part). **b** The presence of amyloid structures after the cell fractionation procedure was assessed with ThT staining. Quantification of the ThT fluorescence in each condition is reported on the histogram (*n* = 3). The average values ± SD are used to present the data. (*) *p* < 0.05, (***) *p* < 0.001. The significance of the ThT fluorescence differences were tested using Student’s *t*-test. Source data are provided as a Source Data file.

Given that GFP-Pga59 colocalized with the ThT signal at the cell wall of *C. albicans* upon adhesion, we performed cell fractionation experiments to determine whether Pga59 is used by fungal cells to form amyloid material. We took advantage of the protocol published by Krynduskin *et al*. to extract amyloid structures from *C. albicans*^39^. Cells expressing either untagged Pga59, GFP-Pga59 or GFP-Pga59^V32,33N^ were treated with BSA-coated magnetic beads to trigger the adhesion process. Cells which had not been treated with magnetic beads were used as a negative control as we had previously shown that Pga59 amyloid production was almost absent in non-adherent cells. Cells from the different strains and adhesion conditions were successively: (i) disrupted with glass beads, (ii) subjected to ultracentrifugation on a sucrose pad to enrich for amyloids and (iii) the resulting amyloids structures were submitted to an immunogold labelling procedure and the samples observed with a transmission electron microscope. As expected, no amyloid was found in samples obtained from non-adherent cells (Fig. 5a). However, amyloid structures were observed in the samples from both adherent WT cells and GFP-Pga59 expressing strains (Fig. 5a, upper and middle panels, respectively). No amyloid structure was observed on material extracted from adherent cells expressing *GFP-pga59*^*V32,33N*^ (Fig. 5a, lower panels). All samples were submitted to immunolabelling with gold-labelled anti-GFP antibodies. A fraction of the amyloid materials extracted from cells expressing the *GFP-PGA59* allele was positive to the immunogold labelling (black dots of 10 nm on the amyloid structures), which indicates that GFP-Pga59 was used by *C. albicans* to assemble these amyloid structures (Fig. 5a, middle panels). The specificity of the immunogold labelling was confirmed as no gold particles were associated with amyloid structures extracted from the WT strain expressing the untagged version of Pga59 (Fig. 5a, upper panels). To verify that aggregates resulting from the cell fractionation are genuine amyloids, we stained each protein sample with Thioflavin T. Extracts from adherent cells expressing either the untagged or the GFP-tagged version of Pga59 yielded a ThT fluorescence signal of 2000 AU and 1900 AU respectively, while mixtures coming from either non-adherent cells or cells expressing the GFP-tagged mutant version of Pga59 presented a weak ThT signal (Fig. 5b). Taken together, these results reveal that Pga59 is a component of the amyloid structures crafted by *C. albicans* in response to adhesion. The data also pinpoints the fact that replacing valine residues at positions 32 and 33 with asparagines tremendously reduces the ability of the fungus to produce adhesion-triggered amyloids.

### The amyloid property of Pga59 positively impacts adhesion and biofilm formation in *C. albicans*

**Fig. 6:**
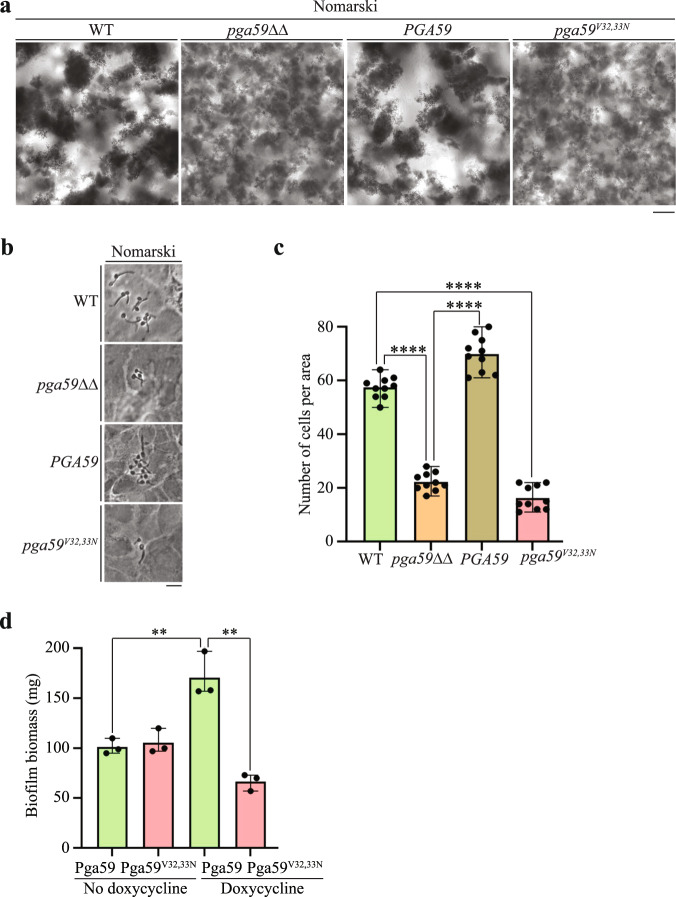
Pga59^V32,33N^ impairs adhesion on biotic and abiotic surfaces as well as biofilm establishment in *C. albicans*. **a** Adhesion to abiotic surfaces was assessed by cultivating the indicated strains with magnetic beads. The formation of cellular aggregates was assessed by transmission microscopy (Nomarski). Scale bar: 200 μm. **b** The indicated strains were cultivated on a monolayer of Caco-2 cells to monitor adhesion on biotic surfaces. After 1 h of adhesion, *C. albicans* cells were washed and observed under the microscope (Nomarski). Scale bar: 10 μm. **c** *C. albicans* adhered on Caco-2 cells were counted on 200 µm^2^ areas. The experiment was repeated three times. Significance was assessed using Student’s *t*-test. (****) *p* < 0.0001. **d** Biofilms of the indicated overexpression strains were grown in a continuous flow microfermentor in a GHAUM medium in the presence or absence (negative control) of 50 μg mL^−1^ doxycycline. After 40 h, the biofilms were detached from the spatula, dried for 24 h, and weighed. The histogram presents the quantification of data derived from three independent experiments. Values are the average of each condition ± SD. (**) *p* < 0.01. The significance of the dry mass differences between the conditions was assessed using Student’s *t*-test. Source data are provided as a Source Data file.

Proteins with amyloid properties have been described in the literature to be involved in the adhesion process between microbial cells and their substrate^25^. To evaluate if Pga59 is used by *C. albicans* to trigger a cell-substrate adhesion mechanism, we performed adhesion assays on both magnetic beads and Caco-2 cells. Firstly, mid-logarithmic cells were incubated with BSA-coated magnetic beads and then the size of cellular aggregates was recorded with an Opera Phenix microscope. The WT strain was able to form huge cell aggregates tens of micrometres wide (Fig. 6a). Yeast cell aggregates came in the form of irregularly contoured dark structures in the focal plane, surrounded by empty areas (light grey). The size of cell aggregates formed by the *pga59Δ/pga59Δ* strain was greatly reduced, suggesting that adhesion forces between cells were weaker (Fig. 6a). Complementing the KO mutant with the WT *PGA59* allele restored its ability to form huge cell aggregates. Interestingly, cells expressing the mutant *pga59*^*V32,33N*^ in which amyloid assembly is blocked behaved like the KO strain and produced very small cell aggregates (Fig. 6a). We then performed adhesion assays on Caco-2 cells with the same set of strains. A monolayer of Caco-2 cells was infected by *C. albicans*, and after the adhesion time, non-adherent cells were washed away, and adherent cells were counted. Representative pictures of *C. albicans* adhered at the surface of the Caco-2 monolayer (forming germ tubes and filaments) are shown in Fig. 6b. On average, 58 ± 6 cells per area have been counted in the WT context. This number significantly dropped to 21 ± 5 and 19 ± 5 in the KO mutant or the strain expressing the *pga59*^*V32,33N*^ allele respectively. In cells complemented with the WT allele, the average number of cells per area was determined to be 66 ± 10. These results clearly show that Pga59 full amyloid capabilities are required for cells to properly adhere to biotic surfaces.

The involvement of Pga59 in biofilm formation has already been shown. Indeed, in a continuous flow microfermentor model, overexpression of Pga59 was shown to lead to thicker biofilms than the wild type^35^. In the same experimental setting, the *pga59* knock-out strain forms a biofilm twice lighter than that formed by cells expressing Pga59 (Supplementary Fig. 4), thus confirming the implication of Pga59 in this process. To investigate the impact of Pga59 amyloid properties on biofilm establishment, we assessed the biofilm formation capacity in a continuous flow fermentor system of strains overexpressing either *PGA59* or *pga59*^*V32,33N*^. Inducing the overexpression of *PGA59* led to a significantly higher dry biofilm biomass (170 ± 27 mg) as compared to the same strain in uninduced conditions (100 ± 9 mg) (Fig. 6c). In contrast, when *pga59*^*V32,33N*^ was overexpressed, the dry biofilm biomass dropped to 67 ± 6 mg compared to the identical strain without overexpression of *pga59*^*V32,33N*^ (105 ± 15 mg) (Fig. 6c). The overexpression of *PGA59* is associated to an increase of 70% in fungal biofilm production while the overexpression of *pga59*^*V32,33N*^ is linked to a decrease of 37% of the biofilm biomass both compared to strains without overexpression of *PGA59* and *pga59*^*V32,33N*^ respectively.

Overall, these results show that amyloid properties of Pga59 are important for adhesion to both abiotic surfaces and human cells, as well as biofilm establishment in *C. albicans*.

### The Pga59-derived D1 peptide containing the V32,33N mutation impairs amyloid formation upon adhesion and biofilm formation

**Fig. 7:**
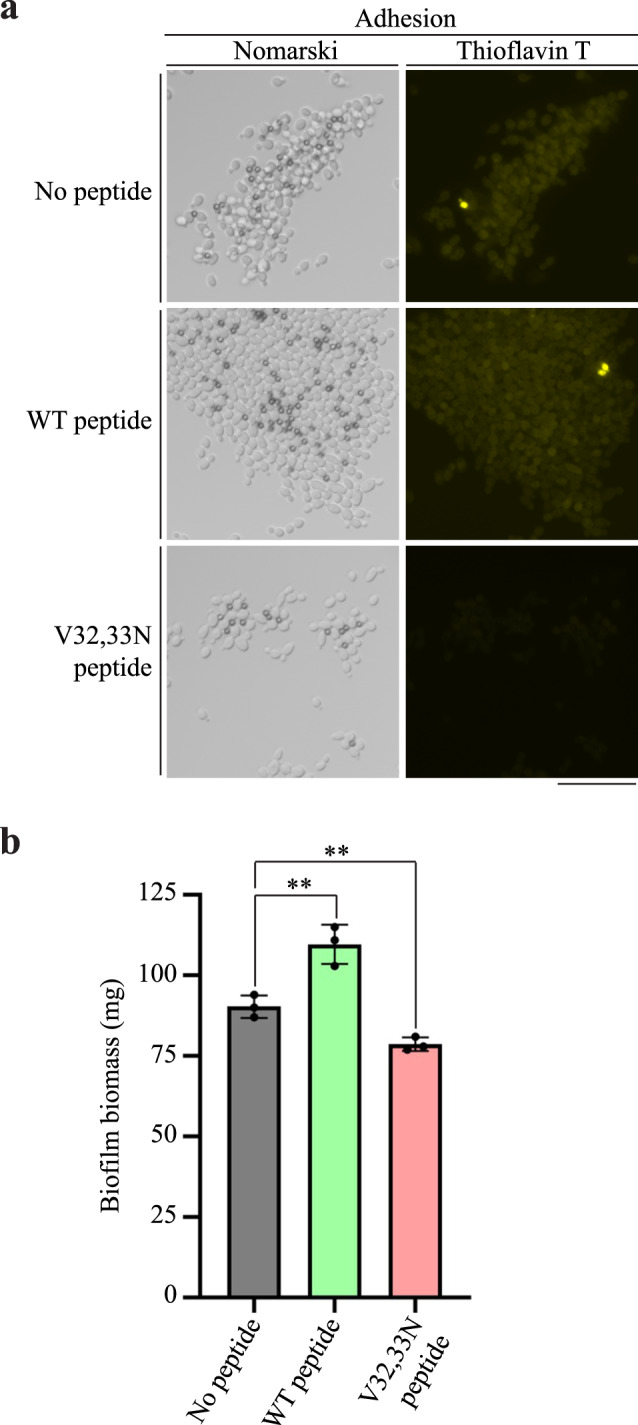
Amyloid assembly and biofilm formation are impaired upon treatment with the Pga59 D1 V32,33N peptide. **a** Amyloid assembly upon adhesion was assessed by incubating a WT strain of *C. albicans* with magnetic beads, ThT and either the Pga59 WT D1 peptide (middle panels) or the V32,33N mutant version (bottom panels). Adherent cells without peptide treatment were used as a control (top panels). The ThT fluorescence was observed with an epifluorescence microscope. Scale bar: 25 μm. The experiment was performed three times. **b** Biofilms of the WT *C. albicans* strain were grown in TPP^®^ six-well culture plates in RPMI medium in the presence of either the Pga59 WT peptide or the V32,33N peptide. Biofilms incubated without Pga59 peptides were used as a control. After 48 h, biofilms were washed, dried, and subsequently weighed. For each experimental condition, values are average ± SD. (**) *p* < 0.01. The quantification of three independent experiments is presented on the histogram. The significance of the results was evaluated using a Student’s *t*-test. Source data are provided as a Source Data file.

Since the V32,33N mutation in the D1 domain of Pga59 impairs amyloid assembly in adherent cells and during biofilm establishment, we hypothesised that treating WT cells with the Pga59 peptide harbouring the V32,33N mutation would impact these biological phenomena. To assess the effect of the peptide on adhesion, 5 μM of either the WT or the mutant version of the D1 Pga59 peptide were added to cells adhered to BSA-coated magnetic beads together with 25 μM of ThT and incubated for 1 h. No peptide was added to ThT in the negative control. Then, amyloid assembly was monitored in each condition by visualisation of ThT staining with an epifluorescence microscope (Fig. 7a). Upon addition of the WT peptide, *C. albicans* formed aggregates even bigger than in the untreated condition. In contrast, the mutant peptide reduced the size of cellular aggregates suggesting reduced adhesion forces between cells compared to untreated cells or cells treated with the WT D1 peptide (Fig. 7a). Amyloid structures were produced to the same extent in both untreated conditions and cells treated with the WT D1 peptide (Fig. 7a). However, yeasts treated with the V32,33N peptide exhibited a weak ThT signal upon adhesion. This result clearly shows that amyloid formation was impaired when adherent cells were incubated with the mutant peptide. As the V32,33N peptide reduced the amyloid assembly and aggregation capabilities of adherent cells, we then tested the effect of this peptide on biofilm formation in TPP^®^ six-well culture plates. About 5 μM of either the WT or the mutant version of the D1 Pga59 peptide were added to the wells after the adhesion step, and the biofilms were allowed to form for 48 h at 37 °C. Incubation with the WT peptide resulted in increased biofilm biomass (109.7 mg) compared to untreated cells (90.33 mg, Fig. 7b). Interestingly, the biofilm biomass dropped significantly to 78.67 mg when cells were treated with the V32,33N peptide (Fig. 7b). Overall, the V32,33N peptide reduces amyloid assembly upon adhesion, and hence impacts biofilm establishment by lowering the biomass of the fungal community.

## Discussion

In the present work, we have investigated the molecular mechanisms underlying Pga59 function regarding biofilm formation in *C. albicans*. Pga59 belongs to the Hwp1 family of adhesins comprised of 12 proteins characterised by a conserved 42 amino acid-long region, the molecular function of which has not been uncovered yet^23,34^. Results from several members of this family, namely Hwp1, Hwp2, Eap1 and Rbt1 have revealed the presence of amyloid-forming regions within the 42 amino acid domains, suggesting a role of this specific domain in amyloid formation^20,40^.

Here, an in silico analysis revealed that the Pga59 42 amino acid domain contains three potential amyloid-forming regions, only one of which exhibits β-aggregation potential. Using an in vitro approach, we demonstrated that Pga59 can be assembled into β-amyloid fibres. Furthermore, we showed that this phenomenon is relevant in cellulo where Pga59-dependent amyloid structures are crafted upon adhesion. Importantly, we also demonstrated that amyloid structures assembled in a Pga59-dependent manner is important for fungal cell adhesion and effective biofilm formation.

Over the past decade, functional amyloids involved in adhesion mechanisms have been described in microorganisms. For instance, the Fap and Sbp amyloid systems, from *Pseudomonas aeruginosa* and *Staphylococcus epidermidis*, respectively, are ECM residents and are involved in cell–cell interactions for biofilm establishment^41,42^. Recently, the Bap system from *Staphylococcus aureus* was also described to trigger cellular adhesion and positively impact biofilm formation^43^. Under acidic pH and low calcium concentration, Bap is released from the cell wall and assembles as an amyloid-like aggregate to stimulate the formation of bacterial biofilm^43,44^. Other examples include *C. albicans* Als1 and Als5 adhesins that are clustered in specific regions of the cell wall, called amyloid nanodomains, to support cell–cell interactions. Blocking amyloid nanodomains assembly by expressing an Als1 mutant unable to form amyloid structures results in reduced cell–cell interactions^45^. Als1 and Als5 are large proteins with a common architecture: an N-terminal Ig-like domain which provides adhesion capabilities to a substrate, a Thr-rich T domain containing the amyloid-forming region required to form the amyloid structures, a TR domain that generates hydrophobic interactions between adhesins and substrates, a stalk region, and a C-terminal GPI anchor^23^. These domains cooperate to establish amyloid nanodomains at the yeast cell surface, while adhesiveness is provided by the Ig-like and the TR domains. Pga59 is a protein about ten times smaller than Als adhesins and shares no sequence similarity with Als proteins except in the amyloid-forming regions. These topological differences suggest that although Als proteins and Pga59 are involved in adhesion processes, they rely on different molecular mechanisms. Moreover, regarding the Hwp1 family of adhesins, Hwp1, Hwp2 and Rbt1 have been implicated in cell–cell aggregation and biofilm formation. While the amyloid-forming regions are conserved between Pga59, Hwp1, Hwp2 and Rbt1, the effector domains allowing Hwp1, Hwp2 and Rbt1 adhesins to adhere on a surface are not conserved in Pga59, making its adhesion mechanism unclear^23^. Further work is needed to decipher the adhesion mechanism of Pga59 to its substrate.

Several adhesins have already been described in the literature as having an impact on biofilm formation in *C. albicans*^23^. Therefore, the relevance of Pga59 in relation to other adhesins in biofilm formation must be discussed. Biofilm establishment in *C. albicans* is a biological process that includes four steps: (i) the adhesion of yeast cells to a surface, (ii) the initiation step where yeast cells start to filament, (iii) the maturation phase to constitute an intricate network of yeast cells encased within an ECM and finally (iv) the dispersion to release yeast cells from the biofilm and colonise new area^46^. Als1 is under the control of the Efg1 transcription factor and hence its expression occurs during filamentation at the maturation step^47^. Moreover, immunofluorescence microscopy clearly shows that Als1 is mainly located at the hyphae cell wall^47^. Hwp1, Hwp2 and Als3 are hyphae-specific proteins expressed once the filamentation is engaged during the maturation step^20,48,49^. Thus, in a fungal biofilm context, these adhesins were suggested to maintain microbial community cohesion by making cell–cell connections. However, in the present report, we showed that adhesion properties provided by Pga59 through cell surface amyloid formation start to occur prior to filamentation (Fig. 4). It is thus suggested that amyloid structures crafted by using Pga59 contribute to the initial adhesion of fungal cells to the substrate which chronologically would happen before the intervention of Als1, Hwp1, Hwp2 and Als3 adhesins. Further studies will be required to validate this statement.

To date, Als1 and Als5 are the only proteins described in *C. albicans* to form amyloid nanodomains and trigger adhesion in response to shear forces generated by a liquid flow^31^. Yet, the fact that Pga59 colocalized with amyloid structures upon adhesion leaves open the possibility for the existence of amyloid nanodomains constituted of Pga59 adhesins (Fig. 4). The β-aggregation-prone amyloid core sequence IVIVATT from Als1 is the minimal sequence to trigger amyloid links between homologous adhesins^45^. Here we showed that the IATTVVTI octapeptide from Pga59 is necessary and sufficient to form fibrillar structures^24,25^. Although the amyloid core sequences of Als1 and Pga59 are different, they are mainly comprised of isoleucines, threonines and valines, which are the residues most often encountered in amyloid-forming cores^24,25^. Therefore, the similar biochemical properties of both sequences suggest that Pga59 could behave as Als1 and generate amyloid nanodomains upon adhesion.

Amyloid structures extracted from adherent *C. albicans* are crafted in a Pga59-dependent manner and contain Pga59. In vitro, amyloid fibres are made up of several hundred monomers of the same protein^50^. However, in bacteria, some functional amyloids incorporate several proteins. For instance, the curli fibre of *E. coli* is mainly composed of CsgA but also includes the minor curli subunit CsgB^51,52^. Furthermore, a fibrous structure produced within the ECM of *Streptococcus mutans* biofilms is positive to immunogold labelling using antibodies targeting WapA and SMU_63c, suggesting that both proteins are assembled in an amyloid aggregate by the bacteria^53^. To date, the protein composition of Pga59-dependent amyloid structures found in the *C. albicans* cell wall upon adhesion is still unclear. The behaviour of the *pga59* knock-out strain, as well as the strain producing Pga59^V32,33N^, let us suggest that Pga59 is not the only protein found in cell surface amyloids of adherent cells. Indeed, although the loss of either Pga59 itself or its ability to form amyloid structures greatly reduced the ThT staining on adherent cells, a weak ThT signal persisted (Fig. 4). Amyloid nanodomains are thought to be exclusively constituted by adhesins homotypic interactions. However, most of the work performed on amyloid nanodomains relied on heterologous expression of *C. albicans* Als adhesins in *S. cerevisiae*. Thus, the composition of amyloid nanodomains in the *C. albicans* cell wall is still not fully elucidated and it is thus possible that Pga59 is assembled within amyloid nanodomains with other adhesins at the cell surface of *C. albicans* upon adhesion.

The assembly of amyloid-like materials in the ECM is a common mechanism used by bacterial species (i.e., *E. coli*, *P. aeruginosa*) to form a biofilm. Here, we have demonstrated that the formation of amyloid materials positively impacts biofilm establishment in *C. albicans*. Cells expressing Pga59^V32,33N^, a mutant version of Pga59 unable to form Pga59-dependent amyloid structures in vivo, produce significantly thinner biofilms compared to a WT strain. Using atomic force microscopy, we had previously shown that overexpression of Pga59 is associated with higher adhesion forces^35^. Altogether, these results let us speculate on a model where Pga59 assembled as an amyloid structure creates adhesion forces which are beneficial for biofilm establishment.

Amyloid structures are directly involved in the pathophysiology of several human diseases^54^. Anti-amyloid compounds inhibiting the assembly of amyloid fibres are thought to be effective molecules to fight diseases where amyloid structures contribute to the pathology^55^. Regarding fungal biofilms, an amyloid inhibitory peptide targeting the amyloid-forming region of Als5 was previously shown to greatly reduce *C. albicans* biofilm formation in vitro^38^. Cells expressing *pga59*^*V32,33N*^ showed a tremendous reduction in their abilities to both produce amyloid structures and to adhere on surfaces compared to strains expressing the wild-type version of *PGA59* (Figs. 4–6). Adherent cells treated with the V32,33N mutant peptide showed reduced amyloid formation as well as smaller cellular aggregates compared to *C. albicans* cells treated with the Pga59 WT peptide (Fig. 7a). Additionally, biofilm biomass was lowered when cells were incubated with the Pga59 mutant peptide compared to untreated cells or cells cultivated with the Pga59 WT peptide (Fig. 7b). Adding the mutant peptide weakens adhesion forces between cells most probably by reducing amyloid assembly upon adhesion, and this negatively impacts biofilm formation. Thus Pga59-based peptides harbouring the V32,33N mutation could constitute a promising avenue to develop new antifungal strategies by lowering amyloid assembly and thus impairing the adhesion process and biofilm formation. Further, as Als5-derived peptides have been shown to inhibit cell–cell adhesion^38^, blocking amyloid assembly with a combination of peptides targeting several amyloid proteins important for adhesion could lead to a stronger strategy to block biofilm establishment of the pathogenic yeast *C. albicans*.

## Methods

### Yeast strains and culture conditions

*C. albicans* and *S. cerevisiae* strains used in this study are listed in Table 1. Strains were routinely cultivated in YPD medium (1% yeast extract, 2% peptone and 2% glucose) at 30 °C or SD minimal medium (0.67% yeast nitrogen base without amino acids, 2% glucose) with supplements (arginine 20 μg mL^−1^, uridine 40 μg mL^−1^, histidine 20 μg mL^−1^). *C. albicans* strains were transformed using the lithium acetate-PEG protocol^56^. Briefly, an overnight culture was diluted to OD_600_ = 0.2 in 50 mL YPD and grown at 30 °C until OD_600_ = 0.6–0.8. Cells were rinsed first in ice-cold 10X TE (100 mM Tris pH 7.5/10 mM EDTA), then in ice-cold 1X TE/100 mM lithium acetate, resuspended in 200 μL 1x TE/100 mM lithium acetate and incubated for 1 h on ice; 50 μL of competent cells were mixed with 1 μg DNA, 50 μg of salmon-sperm DNA and 300 μL of a 40% PEG 3000/1X TE/100 mM lithium acetate solution. The transformation mix was then incubated O/N at 30 °C; cells were then heat-shocked for 15 min at 44 °C and washed with 500 μL of SD medium; cells were pelleted by centrifugation and resuspended either in 300 μL of SD medium and plated on selection plates for prototrophic selection, or in 1 mL YPD, the cells incubated at 30 °C for 3–4 h before plating on YPD medium supplemented with 200 μg mL^−1^ nourseothricin. *S. cerevisiae* strains were routinely transformed with the lithium method^57^. In short, cells were grown as described above for *C. albicans*, but rinsed in water and resuspended in 1 mL of water. One hundred μL of the cells were used per transformation as follows: cells were pelleted by centrifugation and resuspended in 360 μL transformation mix (30% PEG 3000; 100 mM LiAc, 50 µg salmon-sperm DNA; up to 1 μg DNA). Cells were heat-shocked at 42 °C for 40 min, pelleted, resuspended in 300 μL of water and plated on SC-Ura medium (0.67% yeast nitrogen base without amino acids, Dropout mix without uracil and 2% glucose). For biofilm formation in a continuous flow fermentor system, *C. albicans* cells were cultivated in GHAUM medium (SD minimal medium supplemented with arginine 20 μg mL^−1^, uridine 40 μg mL^−1^, histidine 20 μg mL^−1^ and methionine 40 μg mL^−1^) with or without 50 μg mL^−1^ of doxycycline.

**Table 1:** Strains used in this study.

Strain	Parent	Genotype	Reference
*C. albicans*			
BWP17		*ura3::λimm434/ura3::λimm434 arg4::hisG/arg4::hisG his1::hisG/his1::hisG*	^63^
CAI4		*ura3::λimm434/ura3::λimm434*	^64^
CEC175	BWP17	*ura3::λimm434/URA3 arg4::hisG/arg4::hisG his1::hisG/his1::hisG*	Lab’s collection
CEC369	CEC175	*ura3::λimm434/URA3 arg4::hisG/ARG4 his1::hisG/HIS1*	^65^
CEC373	CEC175	*ura3::λimm434/URA3 arg4::hisG/arg4::hisG his1::hisG/his1::hisG pga59∆::HIS1/pga59∆::ARG4*	^66^
CEC832	CEC373	*ura3::λimm434/URA3 arg4::hisG/arg4::hisG his1::hisG/his1::hisG pga59∆::HIS1/pga59∆::ARG4 RPS1/RPS1::*CIpSAT*::PGA59*	^66^
CEC1429	CAI4	*ura3::λimm434/ura3::λimm434 ADH1/adh1::*P_*ADH1*_*-cartTA::SAT1::*P_*TET*_*-caGFP*	^35^
CEC6217	CEC373	*ura3::λimm434/URA3 arg4::hisG/arg4::hisG his1::hisG/his1::hisG pga59∆::HIS1/pga59∆::ARG4 RPS1/RPS1::*CIpSAT*::pga59*^*V32,33N*^	This work
CEC56	BWP17	*ura3::λimm434/ura3::λimm434 arg4::hisG/arg4::hisG his1::hisG/his1::hisG PGA59/PGA59::(GFP-PGA59-ARG4)*	^34^
CEC6218	CEC373	*ura3::λimm434/URA3 arg4:hisG/arg4::hisG his1::hisG/his1::hisG pga59∆::HIS1/pga59∆::ARG4 RPS1/RPS1::*CIpSAT*::GFP-pga59*^*V32,33N*^	This work
CEC6219	CEC1429	*ura3::λimm434/ura3::λimm434 ADH1/adh1::*P_*ADH1*_*-cartTA::SAT1::*P_*TET*_*-caGFP RPS1/RPS1::*CIp10*-*P_*TET*_*-PGA59*	^35^
CEC6220	CEC1429	*ura3::λimm434/ura3::λimm434 ADH1/adh1::*P_*ADH1*_*-cartTA::SAT1::*P_*TET*_*-caGFP RPS1/RPS1::*CIp10*-*P_*TET*_*-pga59*^*V32,33N*^	This work
*S. cerevisiae*			
BY4742		*MATα his3Δ1 leu2Δ0 lys2Δ0 ura3Δ0*	^66^
VIF205	BY4742	BY4742/pBC542(*URA3*)	^40^
CEY679	BY4742	BY4742/pBC542(*URA3* + *PGA59*)	This work
CEY680	BY4742	BY4742/pBC542(*URA3* + *pga59*^*V32,33N*^)	This work

### DNA constructs

Primers used to generate the DNA constructs are listed in Table 2. The mutant ORF *PGA59*^V32,32N^ was created by fusion PCR using CIpSAT1::*PGA59* as a template^34^. Primers A and B and primers C and D were used to amplify two overlapping fragments with the Q5 High-Fidelity DNA polymerase (NEB) (98 °C-30s; (98 °C-10 s; 59 °C-45 s; 72 °C-60 s) x36; 72 °C-2 min), and the fusion PCR (98 °C-30 s; (98 °C-10 s; 59 °C-45 s; 72 °C-90 s) x36; 72 °C-2 min) was performed by using 300 ng of each fragment as templates and primers A and D. Similarly, a PCR fusion was performed to engineer the *GFP*-tagged *PGA59*^*V32,32N*^ mutant using pGEM-T::(*PGA59-GFP*, *ARG4*) as a template^34^; two overlapping fragments were obtained with primers E and B and primers F and C, respectively, using the Q5 High-Fidelity DNA polymerase as above. The fusion PCR was performed as before, using primers E and H. Then, *PGA59*^*V32,32N*^ and *GFP*-*PGA59*^*V32,32N*^ were cloned in the KpnI and SalI sites of CIpSAT2 to generate CIpSAT2::*PGA59*^*V32,33N*^ and CIpSAT2::*GFP-PGA59*^*V32,33N*^, respectively.

**Table 2:** Oligonucleotides used in this study.

Name	Sequence (5′-3′)
A	GGTACCTCTACAGTAGTAGTAGTAGTA
B	CTTCACAAGAAGTGATGGTATTATTGGTGGTAGCAATGTCAGTGAC
C	GTCACTGACATTGCTACCACCAATAATACCATCACTTCTTGTGAAG
D	GTCGACAACTTGATGCACCTACGCA
E	ATCGGTACCTAGTGATAGTAAGTGGTTGG
F	ATCGTCGACCAGGAGAAACAAAGATGTAT
G	GGGGACAAGTTTGTACAAAAAAGCAGGCTGTACGCTAACTCCACTGTCACTG
H	GGGGACCACTTTGTACAAGAAGCTGGGTCTACCTTCAGCAGTAGAAACTG
pga59F	CACTGACATTGCTACCACCG
pga59R	GGGTGGTGGTGGAGTTAGAA
act1F	TTGGATTCTGGTGATGGTGT
act1R	TGGACAAATGGTTGGTCAAG

For expression of HA_3_-Pga59 and HA_3_-Pga59^V32,33N^ proteins in the cell wall of *S. cerevisiae*, *PGA59* (primers G and H) and *PGA59*^*V32,32N*^ (primers G and H) were PCR-amplified from CIpSAT1::*PGA59* and CIpSAT1::*PGA59*^*V32,33N*^, respectively, with the Q5 High-Fidelity DNA polymerase (98 °C-30 s; (98 °C-10 s; 59 °C-45 s; 72 °C-45 s) x36; 72 °C-2 min). We then used the Gateway recombinase-based cloning system (Thermo Fisher Scientific) to introduce the fragments in pBC542^58^, according to the manufacturer’s instructions. The resulting vectors have been named pBC542(*URA3* + *PGA59*) and pBC542(*URA3* + *PGA59*^*V32,33N*^).

### Prediction of amyloidogenic domains in Pga59 and other related proteins

The amyloidogenic domains in Pga59 full-length primary sequence were predicted using the AMYLPRED2 (http://aias.biol.uoa.gr/AMYLPRED2/) and TANGO (http://tango.crg.es) tools^36,37^. The primary sequence of Pga59 was obtained from Uniprot (https://www.uniprot.org/) with the following accession number: Q5AF39.

### In vitro assembly of fungal amyloid fibres

In order to investigate amyloid fibres formation in vitro, synthetic octapeptides corresponding to the wild type (NH_2_-IATTVVTI-COOH) and mutant versions V32N (NH_2_-IATTNVTI-COOH), V33N (NH_2_-IATTVNTI-COOH) and V32,33N (NH_2_-IATTNNTI-COOH) of the Pga59 D1 domain were purchased from Thermo Fisher Scientific. Peptides were resuspended at 100 μM in DMSO. Next, each peptide was diluted at 5 μM in a buffer composed of 20 mM Tris-HCl pH 7.4, 150 mM NaCl, 40 μM thioflavin T and incubated at 37 °C for 16 h.

For rHIS_6_-Pga59 and rHIS_6_-Pga59^V32,33N^ purified from *Escherichia coli*, 5 μM of recombinant protein was incubated at 37 °C for 16 h in the same solution used to trigger the aggregation of D1 peptides.

### Expression and purification of rPga59

*PGA59* codon-optimised synthetic gene lacking the SP_ER_ (signal peptide) and SP_GPI_ (propeptide used to post-translationally modify a protein with a GPI anchor) coding regions were produced by GeneArt Gene Synthesis (Thermo Fisher Scientific). NdeI and XhoI restriction sites added at the N- and C-terminal ends, respectively, were used to excise the gene from the initial pMAT vector; the fragment was inserted into the pET28a(+) expression vector previously linearised with the same sites and the ligation was transformed into One Shot TOP10 Chemically Competent *E. coli* cells (Thermo Fisher Scientific) according to the manufacturer’s instructions. The pET28a(+)-*PGA59* construct was verified by sequencing. The pET28a(+)-*PGA59*^*V32,33N*^ vector was produced by GeneArt Gene Synthesis (Thermo Fisher Scientific). Both plasmids were transformed in *E. coli* SHuffle® T7 Express lysY Competent cells according to the manufacturer’s instructions, and selected on LB containing 30 mg L^−1^ kanamycin. For HIS_6_-Pga59 and HIS_6_-Pga59^V32,33N^ production, 50 mL of overnight bacterial cultures were inoculated into 2.5 L baffled flasks containing 1 L of LB supplemented with 30 mg L^−1^ of kanamycin. Cultures were grown by shaking at 220 rpm at 30 °C until OD_600_ reached 0.6. *PGA59* expression was induced with 1 mM IPTG at 37 °C for 4 h. Bacterial cells were sedimented by centrifugation at 3315×*g* for 20 min, weighted and stored at −80 °C. Cell pellets were thawed on ice and subsequently resuspended in a 50 mM Tris-HCl pH 8400 mM NaCl, 0.1% Triton X-100, 1 mM DTT lysis buffer supplemented with cOmplete^TM^ Mini EDTA-free protease inhibitor (Roche) at a ratio of 3 mL/g of pellet. One mg mL^−1^ of lysozyme was added to the mixture to allow cell lysis at 4 °C for 45 min. Then, 50 μg mL^−1^ of DNase I was added and lysates were incubated for 45 min at 4 °C, followed by centrifugation at 27,000×*g* for 15 min. The resulting pellets were washed two times for 1 h at 4 °C with 25 mL of lysis buffer containing 1% of Triton X-100 and dissolved overnight at 4 °C with 25 mL of lysis buffer containing 6 M of guanidine hydrochloride. HIS_6_-Pga59 and HIS_6_-Pga59^V32,33N^ polypeptides were then purified on a 1 mL Protino® Ni-NTA column (Macherey-Nagel) using the ${{{\ddot{\mathrm A}}}}$KTA Express chromatography system at 4 °C, and a linear gradient of imidazole to a final concentration of 0.5 M. The presence of both peptides was assessed at different steps of the purification by loading 10 μL of rPga59 onto a 4–20% gradient Criterion™ gel (Bio–Rad), using 10 μL of Multicolour Low Range protein ladder (Euromedex) as a molecular weight marker, and either staining 30 min with InstantBlue® Coomassie protein (Abcam) followed by a water rinse, or transferring onto a PVDF membrane with an iBlot2 (Thermo Fisher Scientific) using programme 0 (step 1: 20 V for 1 min, step 2: 23 V for 4 min, step 3: 25 V for 2 min); the membrane was blocked with 5% skimmed milk in 1x PBS for 1 h at room temperature. After rinsing in 1X PBS, the membrane was incubated for 1 h with the A7058 anti-His-tag antibody (Sigma-Aldrich) diluted 1/1000 in 1x PBS. The membrane was then washed, treated with Clarity^®^ Western ECL Substrate (Bio-Rad) and observed using the ChemiDoc MP imaging system (Bio-Rad). Uncropped and unprocessed scans of the gel and the blot are given in the Source Data file. The fractions that contained rPga59 peptides were dialysed against a 20 mM Tris-HCl pH8, 150 mM NaCl, 4 M guanidine hydrochloride solution supplemented with a glutathione redox couple (10 mM reduced, 1 mM oxidised) using a 3 mL Slide-A-Lyzer G2 Dialysis Cassette, 3.5 K MWCO (Thermo Fischer Scientific). Dialysis was performed at 4 °C overnight under gentle agitation. The dialysis process was repeated 3 times by decreasing the quantity of guanidine hydrochloride from 4 to 0 M. The protein concentrations in the refolded fractions were estimated at 280 nm with a spectrophotometer.

### Thioflavin T assays

Amyloid fibres made either of Pga59 peptides or rHIS_6_-Pga59, or directly extracted from *C. albicans*, were stained with Thioflavin T (ThT), a dye specific for amyloid structures, as follows. Amyloid fibres were diluted (5 μM for peptides and rHIS_6_-Pga59; 100 μg of amyloid mixtures enriched from *C. albicans*) in 300 μL Tris buffer (20 mM Tris-HCl pH8 and 150 mM NaCl) containing 40 μM of ThT (stock solution: 3.3 mM in ethanol). Subsequently, 100 μL of each mixture was deposited in a black flat-bottom 96-wells plate (Greiner) and incubated for 16 h at 37 °C. Then the fluorescence intensity was monitored with a TECAN Infinite 200 Pro spectrophotometer using an excitation wavelength of 440 nm and emission wavelength of 496 nm at 37 °C to evaluate amyloid fibril formation.

For in vivo ThT staining, *C. albicans* strains were grown overnight in a YPD medium at 30 °C. Then, cultures were diluted to an OD_600_ of 0.3 in 4 mL of fresh YPD medium and allowed to grow at 30 °C for an additional 4 h. At the mid-logarithmic phase, *C. albicans* strains were washed twice with sterile PBS and resuspended in 1 mL of SD medium. Subsequently, 10^8^ yeast cells were incubated with 10^6^ magnetic beads treated overnight with 1 mg/mL^−1^ heat-denatured BSA on a benchtop rotator for 1 h to trigger adhesion or left untreated (negative control)^24,59^. Then, all fungal cultures were treated with 25 μM of ThT and incubated at room temperature on a benchtop rotator for 1 h. For testing the effect of WT and mutant peptides on cell adhesion, 5 μM of either NH_2_-IATTVVTI-COOH or NH_2_-IATTVVTI-COOH were added during the incubation with ThT.

*S. cerevisiae* BY4742 strains expressing either the WT allele of Pga59 (CEY679), the Pga59^V32,33N^ mutant (CEY680) or the empty vector (VIF205) were grown in SC-Ura medium at 30 °C. Overnight cultures were diluted in 4 mL of fresh SC-Ura medium to an OD_600_ of 0.5 and incubated with agitation at 30 °C for 4 h. *S. cerevisiae* cultures were centrifuged at 200 × *g* for 3 min and resuspended in 1 mL of SC-Ura medium. Then, cells were simultaneously treated with BSA-coated magnetic beads, 25 μM ThT and 50 μg mL^−1^ Concanavalin A, Alexa Fluor 594 Conjugate. After 1 h of incubation on a benchtop rotator at room temperature, cells were washed two times with SC-Ura medium and observed by epifluorescence microscopy.

Fungal cells (*C. albicans* and *S. cerevisiae*) were observed with an ApoTome microscope (Carl Zeiss) equipped with both Olympus^TM^ 40X X-Apo (0.95 NA) and Olympus^TM^ 100X X-Apo (1.45 NA) objectives. Pictures were recorded with a Nikon DXM1200F digital camera. Thioflavin T signals were recorded with the blue filter, while the Concanavalin A, Alexa Fluor 594 Conjugate fluorescence was recorded with the red filter.

The size of *C. albicans* cellular aggregates in each genetic background upon adhesion was evaluated in Nomarski at a magnification of 20X with the Opera Phenix microscope (Perkin Elmer).

### Negative staining

Ten microlitres of rHIS_6_-Pga59 samples or D1 synthetic peptides were spotted on glow discharged formvar/carbon grids (FCF200-Cu, DELTA microscopies), negatively stained with 2% uranyl acetate, pH 4.2, analyzed with an FEI Tecnai T12 120 kV transmission electron microscope (FEI Company) equipped with a Gatan Ultrascan US4000 CCD detector.

### Immunogold labelling

*C. albicans* cells expressing either *GFP-PGA59* (CEC56), *GFP-pga59*^*V32,33N*^ (CEC6218) or the empty vector (CEC832) were grown in YPD medium. Overnight cultures were diluted to an OD_600_ of 0.3 in 1 L of YPD medium and subsequently incubated under agitation at 30 °C. Yeasts were centrifuged and resuspended in 50 mL of SD medium. Fungal cells were then treated with BSA-coated magnetic beads for 1 h at room temperature to trigger adhesion or left untreated (negative controls). After incubation, cells were centrifuged and stored at −80 °C. Amyloid structures were extracted from *C. albicans* as described by ref. ^39^ Briefly, the pellets were resuspended in 4 mL lysis buffer (50 mM Tris-HCl pH 7.5, 150 mM NaCl, 5 mM MgCl_2_, 0.1% Nonidet P-40, 1 mM PMSF, and a cOmplete protease inhibitor tablet (Roche)); then 600 μL were distributed in ten 1.5 mL screwcap tubes and 200 μL of 0.5 mm glass beads were added. Subsequently, fungal cells were mechanically disrupted six times for 1 min in a Bullet Blender bead beater (Next Advance) at full speed. The whole-cell extract was first incubated for 10 min with 0.1 mg mL^−1^ of RNAse A at room temperature, and then with 0.5% of Triton X-100 at 4 °C for 10 min. Samples were centrifuged at 2000 × *g* for 10 min at 4 °C and supernatants were loaded on a 40% sucrose pad. The protein mixtures were then ultracentrifuged at 200,000 × *g*, 4 °C for 2 h. Pellets were resuspended in the amyloid-prion resuspension buffer (50 mM Tris-HCl pH 7.4, 100 mM NaCl, 2% SDS, 5 mM DTT and 5% glycerol) and loaded onto an acrylamide gel. Following the migration, amyloids were eluted from the gel with Amyloid-prion buffer R (Tris-HCl pH 7.4, DTT 5 mM and SDS 0.4%). Ten microlitres of enriched amyloid structures were spotted on glow discharged formvar/carbon grids and processed throughout an immunogold labelling procedure as described in Mourer et al.^60^. Briefly, electron microscopy grids were incubated for 5 min in a droplet of blocking solution (1X PBS, 1% BSA), and then in a solution of 1X PBS, 1% BSA containing the α-GFP antibody (Santa Cruz, Clone B-2 diluted 200 times) for 1 h. After five washes in 1X PBS, 0.1% BSA and five washes in 1X PBS, grids were incubated for 20 min in a solution composed of 1X PBS, 1% BSA and a 1/50 dilution of 10 nm gold particles conjugated to protein A. Grids were washed several times in 1X PBS and incubated in 1X PBS, 1% glutaraldehyde. Finally, grids were washed with distilled water, incubated with 4% uranyl acetate for 30 min, rinsed five times in a droplet of water, and incubated in 2% lead citrate for 2 min. Grids were then rinsed five times in water and dried for at least 24 h at room temperature. Samples were observed with an FEI Tecnai T12 120 kV transmission electron microscope (FEI Company) equipped with a Gatan Ultrascan US4000 CCD detector.

### Adhesion assay

CEC369 (WT), CEC373 (*pga59Δ/pga59Δ)*, CEC832 (*pga59Δ/pga59Δ* CIpSAT1-*PGA59*) and CEC6217 (*pga59Δ/pga59Δ* CIpSAT1-*PGA59*^*V32,33N*^) were used to test the involvement of Pga59 on cellular adhesion as described in ref. ^61^ as follows. Caco-2 (colon carcinoma) epithelial cells were kindly provided by the Dynamics of Host-Pathogen Interactions Unit, Institut Pasteur. A monolayer of Caco-2 cells was cultivated on coverslips deposited in each well of a TPP® 24-well plate (Merck) in DMEM medium (Gibco) supplemented with 10% FBS for 5 days at 37 °C with 5% CO_2_. Caco-2 cells monolayers were washed with sterile PBS and then infected with 2 × 10^5^ fungal cells per mL diluted in RPMI medium. After 1 h, non-adherent *C. albicans* cells were washed off three times with sterile PBS. The remaining adherent cells were cross-linked to Caco-2 cells by overnight incubation at 4 °C with Histofix^®^. Following the chemical fixation, coverslips were washed with PBS and mounted on a microscope slide with a droplet of ProLong^TM^ Gold antifade (Thermo Fisher Scientific) mounting medium. Fifty random pictures per sample showing an area of 200 μm × 200 μm were recorded in Nomarski on an ApoTome microscope. The number of cells was counted and averaged in each genetic background. The results presented here are representative of three independent experiments. The statistical analysis was performed on GraphPad Prism 9 using a Student’s *t*-test.

### Immunofluorescence

*S. cerevisiae* cells transformed with pBC542_HA_3_-Pga59 (CEY679) pBC542_HA_3_-Pga59^V32,33N^ (CEY680) or pBC524 (VIF205) were grown in SC-Ura medium. About 80 × 10^6^ cells were fixed with 4.6% formaldehyde for 40 min at room temperature, washed two times with PBS and then resuspended in 1 mL of PBS. Twenty microlitres of yeast cells were dropped microscope slides previously coated with 0.1% on poly-l-Lysine (Sigma-Aldrich) and were allowed to adhere to the substrate for 30 min at room temperature. After several washes with 1X PBS, cells were covered with 20 μL of 1X PBS, 0.5% BSA for 5 min. The samples were successively incubated with 20 μL of: (i) a monoclonal α-HA antibody (working dilution:1/1000, Santa Cruz clone F-7) in 1X PBS + 0.5% BSA overnight at 4 °C and then (ii) a secondary α-mouse antibody conjugated to Texas Red-X (working dilution: 1/1000, Invitrogen T-862) in 1X PBS + 0.5% BSA for 1 h at room temperature. A drop of ProLong^TM^ Gold antifade mounting medium and a cover slip were successively added to the samples. *S. cerevisiae* strains were observed with an ApoTome microscope. Cells were visualised with Nomarski and the red channel was used to record the fluorescent signal generated by the secondary antibody.

### Western blot analysis

Whole-cell extracts from both adherent *S. cerevisiae* and *C. albicans* cells were obtained with the trichloroacetic acid (TCA) extraction method. Briefly, 300 × 10^6^ fungal cells were resuspended in 1 mL of 20% TCA and centrifuged for 4 min at 200×*g*. The resulting pellets were resuspended in the lysis buffer (400 μL of 6% TCA, 1 mM of PMSF and 400 μL of 0.1 mm glass beads) prior to their mechanical disruption with a Bullet Blender bead beater (six cycles of 1 min full power with 1 min incubation time on ice between each cycle). Glass beads were discarded by centrifugation (1 min at 960 × *g*), and then, the protein mixture was centrifuged 5 min at 2700 × *g*. Pellets were washed three times with 200 μL acetone and resuspended in a solution of 20 mM Tris-HCl pH8 and 1% SDS. Twenty-five micrograms of proteins from each sample were mixed with 2X Laemmli buffer (Bio-Rad) and loaded on a 10% Criterion™ XT Bis-Tris Precast Gel (Bio-Rad). Ten microlitres of PageRuler™ Plus Prestained Protein Ladder (Thermo Fisher Scientific) was used as a molecular weight marker. Proteins were transferred on a PVDF membrane with an iBlot2 dry blotting system (Thermo Fisher Scientific). The membrane was coloured with 1% (w/v) Ponceau S Red in 5% acetic acid to assess the protein load and rinsed with water. Western blotting using as primary antibodies either a mouse monoclonal α-HA antibody (working dilution: 1/1000, Santa Cruz clone F-7) or a rat monoclonal α-alpha tubulin (working dilution: 1/1000, AbD Serotec MCA78G, clone YL1/2) for *S. cerevisiae* or a mouse monoclonal α-GFP antibody (working dilution: 1/1000, Santa Cruz Clone B-2) for *C. albicans* was performed as follow: PVDF membranes were incubated with primary antibodies in 1X PBS + 1% skimmed milk overnight at 4 °C. They were then washed several times in PBS and incubated with the Peroxidase AffiniPure Goat anti-mouse secondary antibody (working dilution: 1/1000, Jackson ImmunoResearch) or HRP-conjugated Goat anti-rat secondary antibody (working dilution: 1/1000, Invitrogen, 629520) in 1X PBS + 1% skimmed milk for 1 h at room temperature. Membranes were then successively washed in 1X PBS, treated with Clarity^®^ Western ECL Substrate (Bio-Rad) and observed using the ChemiDoc MP imaging system (Bio-Rad). Pictures are representative of three independent experiments.

### RNA extraction and RT-qPCR

About 15 mL of CEC369 (WT), CEC373 (*pga59Δ/pga59Δ*), CEC832 (*pga59Δ/pga59Δ* CIpSAT1-*PGA59*) and CEC6217 (*pga59Δ/pga59Δ* CIpSAT1-*PGA59*^*V32,33N*^) were cultivated as described in the thioflavin T assays section. Total RNAs of adherent cells were extracted with the RNeasy mini kit (Qiagen) as per the manufacturer’s instructions. Five hundred micrograms of total RNA were used for reverse transcription using the QuantiTect Reverse Transcription kit (Qiagen) according to the manufacturer’s specifications. The qPCR reaction mixtures contained 1 μL of the resulting cDNA, 4 μL of primer mix at 10 pmol μL^−1^ (primers reverse and forward primers of the target genes, see Table 2), 5 μL of water and 10 μL of 2X SYBR green mix (Eurobio). Q-PCRs were performed in a CFX96 touch real-time PCR detection system (Bio-Rad) apparatus with the following cycle: 50 °C-2 min; 95 °C-10 min; (95 °C-15 s; 59 °C-1 min) x40. The relative expression level of *PGA59* in each genetic background was determined using specific primers (pga59F and pga59R, Table 2) and compared to the expression level of ACT1 (primers act1F and act1R, Table 2).

### Biofilm formation in a continuous flow fermentor system

Cells overexpressing either *PGA59* (CEC6219) or *PGA59*^*V32,33N*^ (CEC6220) were grown in a YPD medium with or without 50 μg mL^−1^ of doxycycline for 16 h. Each culture was then diluted to an OD_600_ of 1 in fresh GHAUM medium with or without 50 μg mL^−1^ of doxycycline and left at room temperature for 30 min, to allow further overexpression. For each condition, plastic slides (Thermanox; Nunc) were immersed in the diluted cultures for 90 min at room temperature to allow adherence of fungal cells to the plastic substrate. The plastic slides were then transferred to glass vessels (1 vessel per condition). Each vessel has two glass tubes inserted to drive the entry of medium and air, while the used medium is evacuated through a third tube^62^. The flow of the GHAUM medium is controlled by a recirculation pump (Ismatec) set at 0.6 mL min^−1^ and pushed by pressured air supplied at 10^5^ Pa, conditions minimising planktonic phase growth and promoting biofilm formation. The chambers with the plastic substrate were incubated at 37 °C and biofilms were grown for 40 h. Plastic substrates were subsequently immersed in 1X PBS and vortexed to recover the fungal biofilms. The PBS solution containing the biofilms was vacuum filtered through a 1.2 μm filter (Millipore); the filter was dried out at 65 °C for 24 h and then weighed on a precision scale (Mettler AE200; Mettler Toledo) to obtain the dry mass of the biofilm. Three biological replicates were analysed through a Student’s *t-*test.

### Biofilm formation in TPP^®^ 6 well culture plates

*C. albicans* overnight cultures were washed in sterile PBS prior to their dilution at 1 × 10^6^ cells per mL in RPMI medium; then, 6 mL were distributed in TPP^®^ six-well plates and allowed to adhere for 90 min without agitation at 37 °C. Subsequently, non-adherent cells were washed once with sterile PBS and 6 mL of fresh RPMI medium, supplemented with either 5 μM of the WT Pga59 peptide or 5 μM of V32,33N mutant peptide, were added to each well. Fungal cells cultured without peptide were used as control. The plates were covered with BREATHseal™ and biofilms were allowed to form for 48 h at 37 °C under gentle agitation at 110 rpm. Biofilms were then gently washed with sterile PBS and vacuum filtered through a 1.2 μm filter. Filters were dried out at 65 °C for 24 h and then weighed on a precision scale (Mettler AE200; Mettler Toledo) to obtain the dry biofilm biomass.

### X-ray diffraction measurement

Fibre diffraction patterns of fibrils were measured at 4 °C on a Rigaku FRX rotating anode X-ray generator equipped with a Pilatus 200 K hybrid pixel detector (DECTRIS Ltd., Baden-Dättwil, Switzerland) at the copper wavelength. The concentrated hydrated samples were mounted in a MicroLoopsTM from Mitegen (Ithaca, NY, USA) on a goniometer head under the cold nitrogen flow. Each diffraction pattern corresponds to a 360° rotation along the phi axis with an exposure time of 720 s after subtraction of a ‘blank’ image of the same exposure time with only the loop on the goniometer head.

### MALDI-TOF mass spectrometry

His_6_-Pga59 and His_6_-Pga59^V32,33N^ integrity and purity were analysed on a Bruker UltrafeXtreme MALDI-TOF/TOF instrument. A volume of 15 μL of protein was passed through a ZipTip C4 and eluted on an MTP 384 ground steel target plate (Bruker-Daltonics, Germany) with 2 μL of 20 mg mL^−1^ α-Cyano-4-hydroxycinnamic acid in 50% acetonitrile, 0.1% trifluoroacetic acid as matrix solution. Data were acquired using Flexcontrol software (Bruker-Daltonics, Germany) and shots were recorded in positive ion linear mode. Mass spectra were externally calibrated in the m/z range of 5–20 kDa with Protein I (Bruker-Daltonics, Germany) and analysed with the Flexanalysis software (Bruker).

### Reporting summary

Further information on research design is available in the Nature Research Reporting Summary linked to this article.

## Supplementary information

Supplementary figures

Reporting Summary

## Data Availability

The data that support the findings of this study are included in the article, its supplementary information files, or are available from the corresponding authors upon reasonable request.
