# Supplementary material for: The Pga59 cell wall protein is an amyloid forming protein involved in adhesion and biofilm establishment in the pathogenic yeast Candida albicans
Source: NPJ Biofilms Microbiomes. 2023 Jan 25;9:6. doi: 10.1038/s41522-023-00371-x (PMC9877000; doi:10.1038/s41522-023-00371-x)
Supplement: Supplementary file 1 — Supplementary figures [file 41522_2023_371_MOESM1_ESM.pdf]

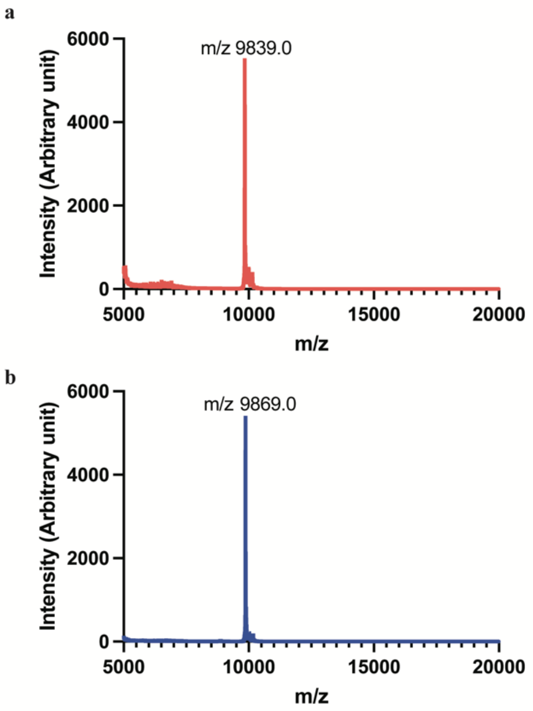

Supplementary Figure 1. Mass spectrometry of Pga59 recombinant proteins.
Recombinant His$_6$-Pga59 (**a**) and His$_6$-Pga59^{V32,33N} (**b**) were purified from *E. coli* inclusion bodies and the mass of both proteins were analyzed by MALDI-TOF mass spectrometry. Source data are provided as a Source Data file.

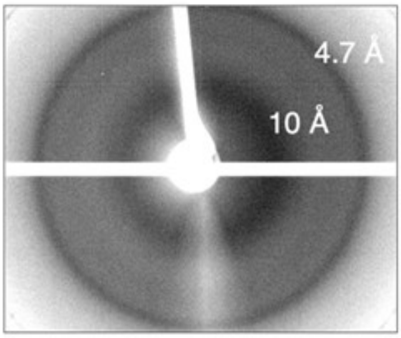

Supplementary Figure 2. His$_6$-Pga59 displays the cross-β diffraction pattern characteristic of β–amyloid structures. His$_6$-Pga59 was expressed in *E. coli* and purified by affinity chromatography using a Ni-NTA column. X-ray diffraction pattern after the aggregation of His$_6$-Pga59 shows reflections at 4.7 Å and 10 Å. The proteins used for X-ray diffraction (Supplementary Fig. 2) or for TEM and ThT staining (Fig 2) come from 2 independent rounds of purification.

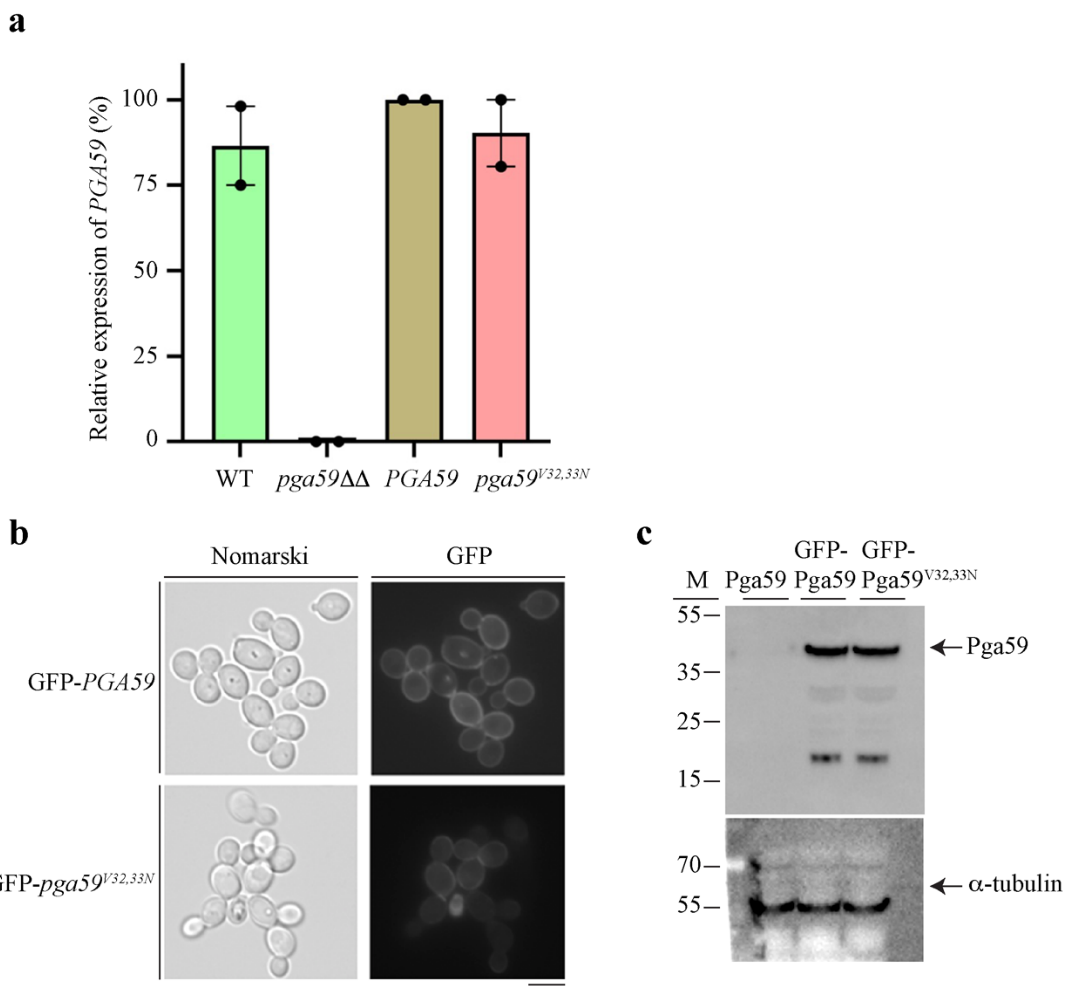

Supplementary Figure 3. Pga59^{V32,33N} is well produced and localized within the cell wall of *C. albicans*. **a**, mRNAs were isolated from *C. albicans* cells treated for 1 hour with BSA-coated magnetic beads to trigger cell-cell adhesion. mRNAs from the indicated strains were used to synthetize cDNAs. *PGA59* gene expression was evaluated using RT-qPCR. The histogram shows the relative expression of *PGA59* in each genetic background. **b**, fungal cells expressing either GFP-Pga59 or GFP-Pga59^{V32,33N} were analyzed by epifluorescence microscopy to assess the cellular localization of the wild type and the mutant versions of Pga59. **c**, whole cell extracts were prepared from adherent cells of *C. albicans* expressing either untagged-Pga59, GFP-Pga59 or GFP-Pga59^{V32,33N}. The quantity of Pga59 was assessed in each strain with an α-GFP antibody while the anti α-tubulin antibody was used as a loading control. Standard molecular weights (M) are indicated on the left. Source data are provided as a Source Data file.

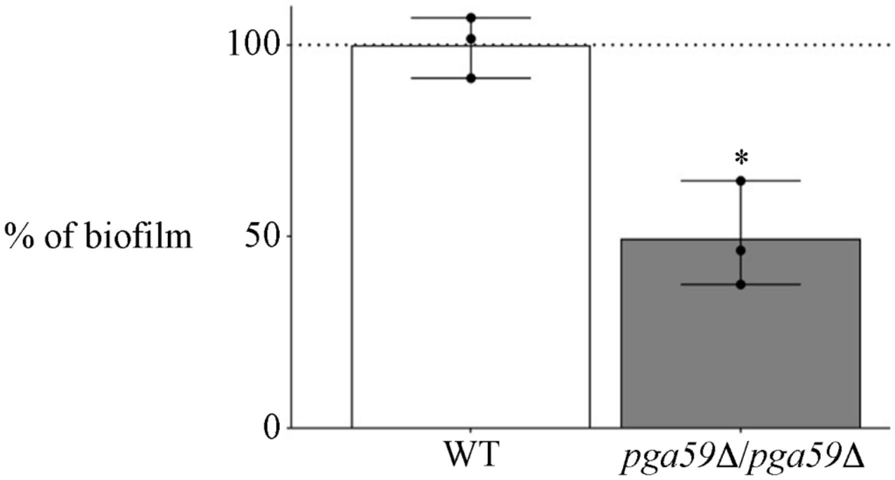

Supplementary Figure 4. *C. albicans* cells lacking Pga59 are defective for biofilm formation. Fungal biofilms were grown in a microfermentor continuous flow system using GHAUM medium. After 40h, the biofilm dry weight was measured in each genetic background. Data come from 3 independent experiments. Significance of the biofilm biomass differences was assessed using a Student's *t*-test. (*) $p < 0.05$. Source data are provided as a Source Data file.
